# Stress Engineering in the Optimization of Next-Generation Hafnium-Based Ferroelectric Memory

**DOI:** 10.3390/nano16090516

**Published:** 2026-04-25

**Authors:** Zhenhai Li, Ruihong Yuan, Xingcan Guo, Yiqun Hu, Yongkai Liu, Jiajie Yu, Kangli Xu, Qingxuan Li, Tianyu Wang, Qingqing Sun, David Wei Zhang, Lin Chen

**Affiliations:** 1 School of Integrated Circuits, Anhui University, Hefei 230601, China; 24041@ahu.edu.cn (Z.L.); huyiqun@ahu.edu.cn (Y.H.); 2 College of Integrated Circuits & Micro-Nano Electronics, Fudan University, Shanghai 200433, China; 23112020045@m.fudan.edu.cn (R.Y.); 24112020065@m.fudan.edu.cn (X.G.);; 3 School of Integrated Circuits, State Key Laboratory of Crystal Materials, Shandong University, Jinan 250100, China; 4 State Key Laboratory of Integrated Chips and Systems, Shanghai 201203, China

**Keywords:** HfAlO thin film, ferroelectric characteristics, first-principle calculations, finite element analysis

## Abstract

Hafnium oxide thin films have been extensively investigated for high-speed and low-power memory applications. Herein, we investigated the influence of oxygen vacancies and external stress on the ferroelectric characteristics of Al-doped HfO_2_ (HfAlO). Compared with HfAlO with 14% oxygen vacancies, films with 21% oxygen vacancies could lower the polarization switching barrier and increase the fraction of the ferroelectric phase. Furthermore, significant external stress promotes ferroelectric phase formation, thereby enhancing ferroelectric characteristics. The remanent polarization achieved with W electrodes (2Pr = 38 µC/cm^2^) is about 18 times that of Au electrodes, owing to the lower thermal expansion coefficient of W electrodes. Density functional theory calculations and finite element analysis provide theoretical insights corroborating the experimental results, helping to pave the way for developing hafnium-based materials for next-generation in-memory computing applications.

## 1. Introduction

With the rapid advancement of artificial intelligence, there is an unprecedented demand for highly efficient information processing in the field of integrated circuits [1,2,3]. Consequently, the development of high-performance memory devices has emerged as a foundational innovation frontier for next-generation information technologies [4,5,6]. Among the candidates for next-generation memory, ferroelectric memory has gained significant attention due to its high-speed operation and low power consumption [7,8,9]. Conventional ferroelectric materials exhibit severe limitations, including heavy metal toxicity, incompatibility with standard CMOS processes, and complex integration schemes [10,11,12]. The discovery of ferroelectricity in hafnium-based materials in 2011 fundamentally addressed these issues [13]. However, HfO_2_-based ferroelectric materials still face challenges, such as the elusive modulation mechanisms of their ferroelectric properties.

Recent studies have shown that oxygen defects have a significant impact on device performance [14,15]. For example, Islamov et al. observed that a wake-up phase is required to increase the remanent polarization at low oxygen vacancy densities, while excessive oxygen vacancies will suppress the ferroelectricity in hafnium-based films [16]. Huang et al. demonstrated that interfacial oxygen injection could reduce the coercive field of hafnium-based ferroelectric thin films by decreasing the oxygen vacancy concentration [17]. Additionally, out-of-plane stress also affects the ferroelectric characteristics of hafnium-based thin films [18,19]. Liu et al. reported that the hysteresis loop contracts with increasing compressive stress [20]. Song et al. suggested that stress plays a crucial role in optimizing the ferroelectric response [21]. Although the above studies have well illustrated empirical strategies for modulating hafnium-based ferroelectric thin films, the underlying modulation mechanisms remain elusive.

In this work, we systematically investigate the effects of oxygen content and mechanical stress on the ferroelectricity of Al-doped HfO_2_ (HfAlO) and its underlying physical mechanisms. The oxygen content of HfAlO is modulated by alternating the oxygen source during deposition. The oxygen vacancies play a crucial role in determining the polarization behavior and stability of HfO_2_. The stress induced in the films is tuned by employing different top electrodes. Stress can significantly alter the microstructure arrangement of HfO_2_-based thin films. Theoretical analyses are conducted to elucidate the mechanisms governing how oxygen content and stress enhance the ferroelectric characteristics.

## 2. Experimental

Initially, SiO_2_/Si substrates (with a 300 nm-thick SiO_2_ layer) were sequentially cleaned using acetone, absolute ethanol, and deionized water [22]. Subsequently, W bottom electrodes were deposited via physical vapor deposition (PVD). Then, 10 nm-thick aluminum-doped HfO_2_ (HfAlO) thin films were deposited on the W/SiO_2_/Si substrates using atomic layer deposition (ALD). Tetrakis(ethylmethylamino) hafnium (TEMAH) and trimethylaluminum (TMA) were employed as precursors, which were alternately pulsed into the ALD chamber at 250 °C using an N_2_ carrier gas (with a TEMAH:TMA cycle ratio of 34:1). H_2_O or O_2_ plasma was used as the oxidant. For the Al_2_O_3_, the ALD temperature was 280 °C. The ALD pulse and purge time were 2 and 5 s, respectively. The growth rate of the Al_2_O_3_ was 1.05 Å for each cycle. For the HfO_2_, the ALD temperature was 280 °C. The ALD pulse and purge time were 5 and 5 s, respectively. The growth rate of the HfO_2_ was 1 Å for each cycle. Finally, W top electrodes (80 × 80 µm^2^) were patterned via a standard lift-off process. A rapid thermal annealing (RTA) treatment was then performed in a nitrogen atmosphere at 500 °C for 30 s.

Based on density functional theory (DFT) calculations using the Materials Studio software 2019, we evaluate the effect of oxygen vacancy on the ferroelectricity of hafnium-based film. The Perdew–Burke–Ernzerhof (PBE) functional of generalized gradient approximation (GGA) is used to optimize the geometric stricture until the residual force on each atom is smaller than 0.02 eV/Å. The crystal structure is a 2 × 2 × 2 supercell with 96 atoms.

## 3. Results

**Figure 1:**
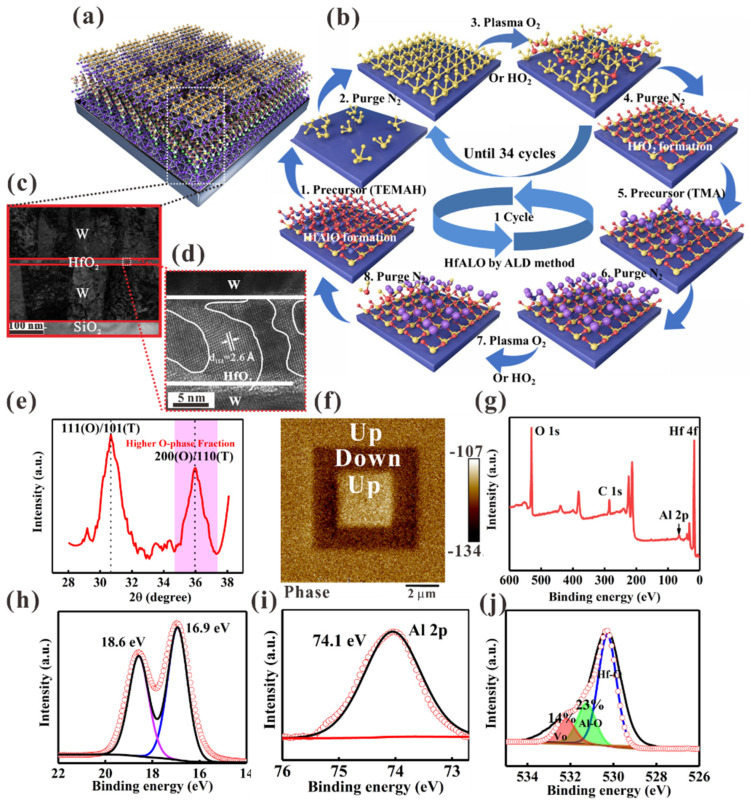
(**a**) Schematic structure of the W/HfAlO/W/SiO_2_/Si devices. (**b**) Schematic of one cycle fabrication process of ALD for HfAlO thin films. (**c**,**d**) The cross-sectional image of the samples and the TEM images of the HfAlO thin films, respectively. (**e**) GIXRD pattern of the HfAlO thin films. (**f**) PFM phase for the HfAlO thin films. (**g**) XPS survey spectra of the initial form of the HfAlO thin films. The coreî−level spectra assigned to (**h**) Hf 4f, (**i**) Al 2p and (**j**) O 1s, respectively.

Figure 1a schematically illustrates the structure of the HfAlO ferroelectric devices. Figure 1b shows that the ALD cycle of the HfAlO thin films consists of 34 cycles of HfO_2_ followed by one cycle of Al_2_O_3_ at a chamber temperature of 250 °C. Cross-sectional scanning electron microscopy (SEM) images reveal distinct interfaces between the device layers, as demonstrated in Figure 1c,d. The thickness of the as-grown HfAlO thin film is approximately 10 nm. Through high-resolution transmission electron microscopy (HRTEM), the HfAlO thin films demonstrate high crystallinity after the RTP process. The measured d-spacing value is approximately 2.6 Å, which is consistent with the X-ray diffraction (XRD) results (Figure 1e). The XRD patterns of the HfAlO film display diffraction peaks at 30.5° and 36°, corresponding to the HfO_2_ orthorhombic (O) (111) and (200) phases, respectively [23,24]. Furthermore, Figure 1f shows that the Piezoelectric Force Microscopy (PFM) response is symmetrical for the +P and –P poled regions with an approximate 27° phase change, indicating robust ferroelectric switching [25,26]. As shown in Figure 1g, the X-ray photoelectron spectroscopy (XPS) survey spectra of the HfAlO films confirm the presence of Hf, Al, and O. Figure 1h shows that the Hf 4f core-level spectrum displays two peaks at 16.9 eV and 18.6 eV, corresponding to Hf 4f_7/2_ and Hf 4f_5/2_, respectively [17,27,28]. Figure 1i shows a characteristic peak located at approximately 74.1 eV, corresponding to the Al 2p core level, which indicates the Al^3+^ chemical state [29]. The O 1s spectrum was deconvoluted into three sub-peaks (Figure 1j). The main peak at approximately 530 eV is attributed to Hf–O bonding, and the 531 eV peak corresponds to Al–O bonding [30,31]. The remaining peak at a higher binding energy is ascribed to the presence of oxygen vacancies, as shown in Figure 1j. Furthermore, the oxygen content of the HfAlO thin films deposited using an O_2_ plasma is higher than that of the films deposited using an H_2_O oxidant, as shown in Figure S1.

**Figure 2:**
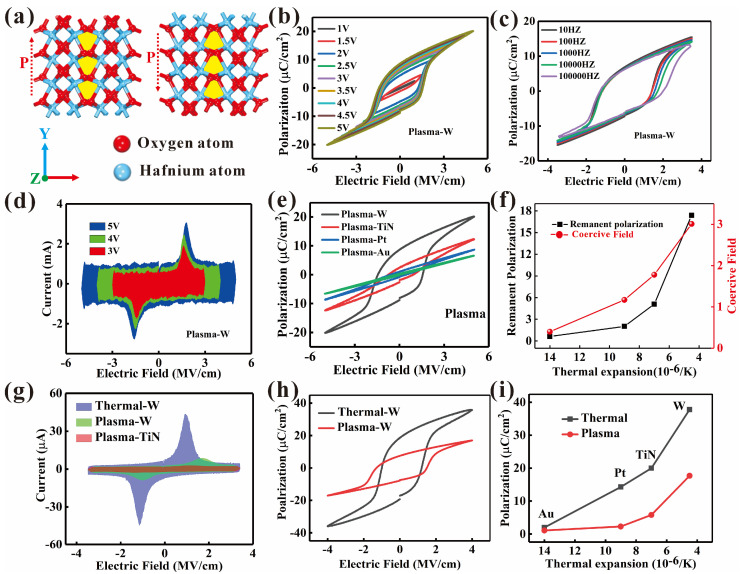
Electrical properties of the HfAlO samples. (**a**) The displacement of O^2−^ ions causes the polarization switching and ferroelectric properties of the HfAlO samples with W top electrodes. (**b**,**c**) The P−E hysteresis curves of the samples at different voltages and frequencies, respectively. (**d**) I−V curves of the HfAlO ferroelectric devices under different voltages. (**e**) The P−E hysteresis curves of the devices with different top electrodes at different voltages. (**f**) The remanent polarization and coercive field change for HfAlO film with 14% oxygen vacancy and changing top electrode. (**g**) I−V curves of the devices with different top electrodes at different voltages. (**h**) The P−E hysteresis curves of the devices with different oxygen sources. (**i**) The effect of the thermal expansion of the top electrode effect on the remanent polarization.

Figure 2a illustrates the atomistic model, where red and blue spheres represent oxygen and hafnium atoms, respectively. As depicted, oxygen atom displacement drives macroscopic polarization switching at room temperature. Figure 2b shows the P-E hysteresis curves of the HfAlO ferroelectric devices under different voltage sweep ranges. As the voltage sweep increases, a larger hysteresis window is obtained. Conversely, Figure 2c demonstrates the degradation of the P-E loops with increasing sweep frequency. As shown in Figure 2d, the current–voltage (I–V) curves exhibit a typical increase in leakage current with applied voltage. Figure S2 displays the capacitance–voltage (C–V) curves measured with bias voltages ranging from −4 V to +4 V at 1 KHz. These collective electrical characterizations indicate robust ferroelectric characteristics and high film quality. Furthermore, we systematically compared the ferroelectric characteristics of devices with different top electrodes and oxygen sources. As shown in Figure 2e, the devices with W top electrodes show well-saturated P-E hysteresis curves, yielding a remanent polarization of approximately 10 µC/cm^2^. In contrast, devices with Au top electrodes display poor P-E hysteresis curves. For the devices fabricated using an H_2_O oxygen source, the P-E hysteresis curves are shown in Figure S3. Figure 2f corresponds to the O_2_ plasma, and shows that both the remanent polarization and coercive field increase as the thermal expansion coefficient (CTE) of the top electrode decreases. For the HfAlO films with an oxygen vacancy concentration of 21.5% corresponding to water source, Figure S4 shows that the remanent polarization similarly increases with decreasing CTE. However, the coercive field exhibits an opposite trend, decreasing with reduced CTE. Figure 2g and Figure S5 show distinct switching current peaks for the devices with W top electrodes. For samples deposited with a H_2_O source, the devices show highly robust P-E curves, as shown in Figure 2h. The devices with HfO_2_ show a high remanent polarization of over 20 µC/cm^2^. From the results, it can be concluded that devices with W top electrodes display superior remanent polarization owing to mechanical confinement provided by their lower thermal expansion coefficient. Additionally, the remanent polarization is further enhanced for devices deposited with an H_2_O source, as summarized in Figure 2i.

**Figure 3:**
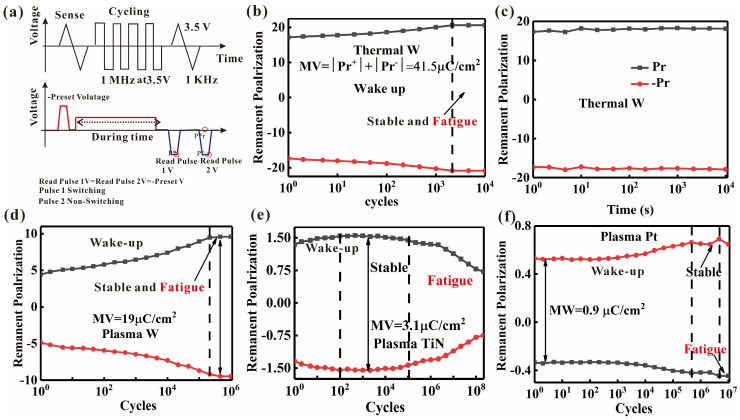
(**a**) Waveform of the endurance of retention characteristics tests. (**b**,**c**) The endurance and retention characteristics of the W/HfAlO/W device with H_2_O as oxygen source, respectively. The endurance characteristics of (**d**) W/HfAlO/W devices, (**e**) TiN/HfAlO/W devices, and (**f**) Pt/HfAlO/W devices with O_2_ plasma as oxygen source.

To systematically investigate the effects of top electrodes and oxygen sources on device characteristics, endurance and retention properties were evaluated as two essential reliability indicators for the HfAlO ferroelectric devices. The applied pulse waveforms for the endurance and retention tests are illustrated in Figure 3a. The endurance characteristics of the devices were tested with a 1000 ns-wide write pulse of 3.5 V and a 1000 ms-wide read pulse of 3.5 V. For the W electrode devices fabricated with an H_2_O oxygen source, Figure 3b shows that the devices exhibit an endurance of over 10^4^ cycles, without significant fatigue degradation. Meanwhile, the retention time is maintained for over 10^4^ s for the ferroelectric devices with W top electrodes, as shown in Figure 3c.

For W electrode devices with an O_2_ plasma source, Figure 3d shows that the devices demonstrate a substantially enhanced endurance of over 10^6^ cycles. Their corresponding retention times are shown in Figure S6a. Notably, compared with the samples derived from H_2_O, the endurance characteristics are improved by two orders of magnitude. Other devices show distinct fatigue behaviors prior to failure. As shown in Figure 3e, the devices with TiN top electrodes achieve an endurance of over 10^8^ cycles, with retention times shown in Figure S6b. The device’s operational lifespan is clearly divided into three distinct stages: wake-up, stabilization, and fatigue. Similarly, the three-stage degradation behavior is also observed for Pt top electrode devices, which yield an endurance of over 10^7^ cycles, as shown in Figure 3f. The underlying physical mechanisms by which the top electrode and oxygen sources modulate the ferroelectric properties will be further analyzed below.

**Figure 4:**
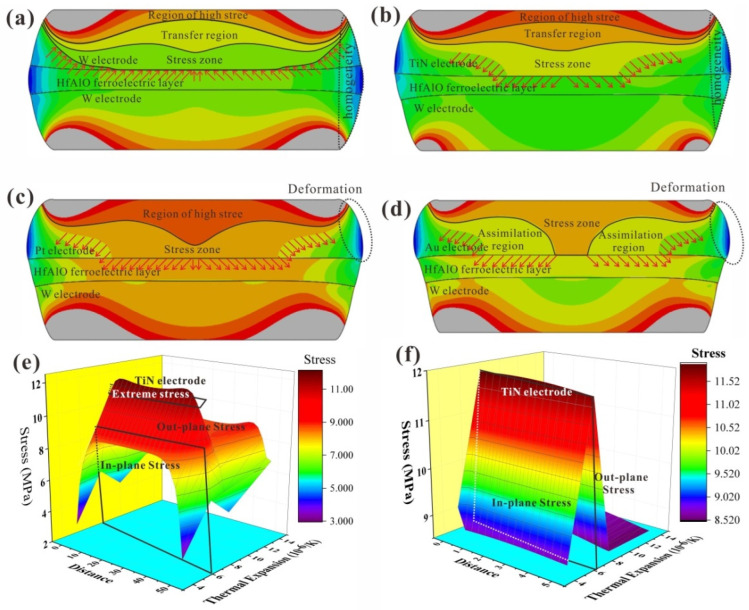
Stress variation in the devices with (**a**) W top electrodes, (**b**) TiN top electrodes, (**c**) Pt top electrodes, and (**d**) Au top electrodes during the annealing process. (**e**) Stress variation in path 3 during the annealing process. (**f**) Stress variation in path 8 during the annealing process.

To elucidate the top electrode effects on the ferroelectric characteristics of the devices, finite element analysis (FEA) was performed using ABAQUS. A two-dimensional model was constructed based on the stacked film structure. Figure 4a shows that the W top electrode can induce large in-plane stress within the HfAlO ferroelectric films owing to the low thermal expansion coefficient (CTE) of metal W. However, other top electrodes predominantly induce out-of-plane stress in the HfO_2_ thin films. As shown in Figure 4b, the TiN electrode applies out-of-plane stress to the ferroelectric thin films, and the stress distribution exhibits three distinct spatial zones: a region of high stress, a transfer region, and a bulk stress zone. Because of the small CTE mismatch between the top electrodes (W as well as TiN) and HfO_2_, no large local deformation occurs during the thermal annealing process, thereby preventing severe stress relaxation. However, Pt and Au electrodes undergo significant deformation during the annealing process due to their high thermal expansion, resulting in pronounced stress dispersion. Figure 4c shows that film deformation may lead to the disappearance of transfer regions. With further increases in the thermal expansion coefficient, Figure 4d shows that the region of high stress disappears, and stress dissipation regions appear. This effectively reduces the stress exerted on the ferroelectric HfAlO films, ultimately leading to the degradation of their ferroelectric characteristics. Figure 4e illustrates that W top electrodes apply in-plane stress to the HfO_2_ thin films, whereas other electrodes exert out-of-plane stress on the functional thin films. Among them, the TiN top electrodes apply the maximum out-of-plane stress to the ferroelectric thin films. A similar stress distribution can also be observed along the vertical depth profile of the films (Figure 4f). Collectively, these simulations elucidate the influence of the electrode thermal expansion coefficient on the ferroelectric properties of the devices.

To better explore the effect of oxygen vacancies on the ferroelectric characteristics of the HfAlO devices, density functional theory (DFT) calculations were performed to determine the polarization switching barrier and the spontaneous polarization of the O-phase in HfO_2_. The DMol3 code within the Materials Studio suite was utilized to elucidate the oxygen vacancy−induced ferroelectric characteristics of HfAlO thin films. A 2 × 2 × 2 supercell with 96 atoms was built for Brillouin zone integration. To obtain an equilibrium structure, geometry optimization was conducted using the Local Density Approximation (LDA) and Projector Augmented Wave (PAW) methods. The structures were optimized until the residual force on each atom was smaller than 0.004 Ha/Å.

**Figure 5:**
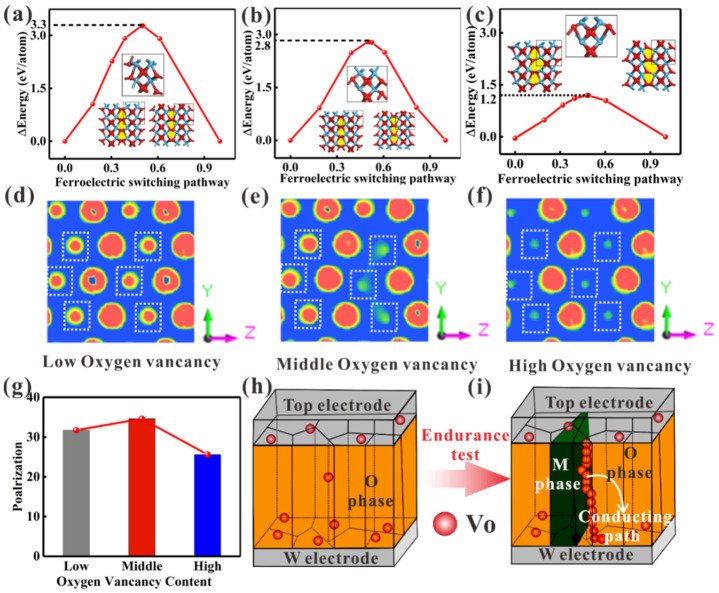
(**a**) The polarization switching barrier of the ideal HfO_2_ cell. The polarization switching barrier of an HfO_2_ cell with oxygen vacancy concentrations of (**b**) 7.6 × 10^21^ cm^−3^, and (**c**) 1.52 × 10^22^ cm^−3^, respectively. (**d**) The isosurface of charge density in the (100) plane for an ideal HfO_2_ cell. The isosurface of charge density in the (100) plane for an HfO_2_ cell with an oxygen vacancy concentration of (**e**) 7.6 × 10^21^ cm^−3^, and (**f**) 1.52 × 10^22^ cm^−3^, respectively. (**g**) The polarization of the O-phase as a function of d100. (**h**,**i**) The schematics of the failure mechanism of the HfAlO devices during endurance cycles.

Figure 5a−c depict the minimum energy paths for switching between the two ferroelectric states, illustrating that the energy barrier is greatly reduced with increasing oxygen vacancy concentration. The switching barrier decreases by 0.5 eV relative to the pristine structure at an oxygen vacancy concentration of 7.6 × 10^21^ cm^−3^ (Figure 5b). At a higher oxygen vacancy concentration of 1.52 × 10^22^ cm^−3^, Figure 5c shows that the switching barrier is further reduced to less than 2.1 eV. The above analysis can explain the macroscopic coercive field reduction owing to the increase in oxygen vacancies.

Meanwhile, the electron density distributions and local geometric effects were investigated. Figure 5d shows slices of the charge density isosurfaces in the (100) plane for the pristine lattice. With oxygen vacancy increase, the electrode density decreases around the hafnium atoms, which may make it easier for oxygen atoms to move, i.e., the reduction in the coercive field (see Figure 5e). As the oxygen vacancies increase, the electron density decreases around the hafnium atoms, which facilitates oxygen atom displacement, i.e., the reduction in the coercive field (Figure 5e). With further increases in oxygen vacancies, the charge density continues to decrease around the hafnium atoms, as shown in Figure 5f, monotonically decreasing the coercive field. However, the HfO_2_ polarization also decreases when the oxygen vacancy concentration reaches 1.52 × 10^22^ cm^−3^ (Figure 5g). Conversely, for HfO_2_ cells with an oxygen vacancy concentration of 7.6 × 10^21^ cm^−3^, the remanent polarization reaches a maximum of 34.8 µC/cm^2^.

Figure 5h illustrates the pristine HfO_2_ cells. With an increase in polarization switching cycles, the O-phase structure may be damaged, leading to M-phase formation and the generation of massive oxygen vacancies. When these oxygen vacancies accumulate in the films, the devices lose efficacy (Figure 5i). For HfO_2_ thin films with excessive oxygen vacancies, the probability of conductive filament formation is higher, resulting in poor fatigue characteristics. Thus, precisely modulating a suitable content of oxygen vacancies is beneficial for optimizing the ferroelectric characteristics of HfO_2_ thin films.

## 4. Conclusions

In this study, we systematically investigated the influence of oxygen content and mechanical stress on the ferroelectricity of HfAlO thin films. The P-E curves demonstrate that an appropriate oxygen vacancy concentration can enhance the polarization properties and decrease the coercive field of HfAlO thin films. Specifically, the HfAlO films with a 21% oxygen vacancy concentration display a remanent polarization of approximately 20 µC/cm^2^. First-principle calculations reveal that an optimal level of oxygen vacancies can lower the polarization switching barrier of the HfO_2_ ferroelectric films and promote the formation of the ferroelectric phase. However, excessive oxygen vacancies degrade the endurance of the thin films (<10^5^ cycles) due to the accelerated generation of defects during cycling. Furthermore, significant external stress can enhance macroscopic film polarization owing to the stabilization of the ferroelectric phase. Our work provides a solid experimental foundation and profound theoretical insights for the optimization of HfAlO ferroelectric thin films, paving the way for their application in next-generation non-volatile memory devices.

## Data Availability

The data that support the findings of this study are available from the corresponding author upon reasonable request.

## Supplementary Materials

The following supporting information can be downloaded at: https://www.mdpi.com/article/10.3390/nano16090516/s1. Figure S1 The core-level spectra assigned to O 1s, the oxygen of hafnium-based thin film form deionized water. Figure S2 C-V curves of the devices at 4 V. Figure S3 The P-E hysteresis curves of the hafnium-based thin films with 21.5% oxygen vacancies. Figure S4 the remanent polarization and coercive field change with the top electrode changing. Figure S5 The P-E hysteresis curves of the hafnium-based thin films with 14% oxygen vacancies. Fig. S6 the retention characteristics test of hafnium-based thin films with 14% oxygen vacancies.
