# Supplementary material for: Stress Engineering in the Optimization of Next-Generation Hafnium-Based Ferroelectric Memory
Source: Nanomaterials (Basel). 2026 Apr 25;16(9):516. doi: 10.3390/nano16090516 (PMC13164971; doi:10.3390/nano16090516)
Supplement: Supplementary file 1 [file nanomaterials-16-00516-s001.zip › nanomaterials-4227359-supplementary.pdf]

nanomaterials

Stress Engineering in the Optimization of Next-Generation Hafnium-Based Ferroelectric Memory

Zhenhai Li [1,†], Ruihong Yuan [2,†], Xingcan Guo [2], Yiqun Hu [1], Yongkai Liu [2], Jiajie Yu [2], Kangli Xu [2], Qingxuan Li [1,*], Tianyu Wang [3,*], Qingqing Sun [2], David Wei Zhang [2] and Lin Chen [2,4,*]

[1] School of Integrated Circuits, Anhui University, Hefei 230601, China; 24041@ahu.edu.cn (Z.L.); huyiqun@ahu.edu.cn (Y.H.)
[2] College of Integrated Circuits & Micro-Nano Electronics, Fudan University, Shanghai 200433, China; 23112020045@m.fudan.edu.cn (R.Y.); 24112020065@m.fudan.edu.cn (X.G.); 21112020017@m.fudan.edu.cn (Y.L.); 22112020127@m.fudan.edu.cn (J.Y.); klxu22@m.fudan.com (K.X.); qqsun@fudan.edu.cn (Q.S.); dwzhang@fudan.edu.cn (D.W.Z.)
[3] School of Integrated Circuits, State Key Laboratory of Crystal Materials, Shandong University, Jinan 250100, China
[4] State Key Laboratory of Integrated Chips and Systems, Shanghai 201203, China
* Correspondence: li. liqx@ahu.edu.cn (Q.L.); tywang@sdu.edu.cn (T.W.); linchen@fudan.edu.cn (L.C.)
† These authors contributed equally to this work.

The supporting information film includes:
FigureS1 The core-level spectra assigned to O 1s, the oxygen of hafnium-based thin film form deionized water
Figure S2 C-V curves of the devices at 4 V
FigureS3 The P-E hysteresis curves of the hafnium-based thin films with 21.5% oxygen vacancies.
Figure S4 the remanent polarization and coercive field change with the top electrode changing.
FigureS5 The P-E hysteresis curves of the hafnium-based thin films with 14% oxygen vacancies.
Figure S6 the retention characteristics test of hafnium-based thin films with 14% oxygen vacancies.

S1 The core-level spectra assigned to O 1s, the oxygen of hafnium-based thin film form deionized water

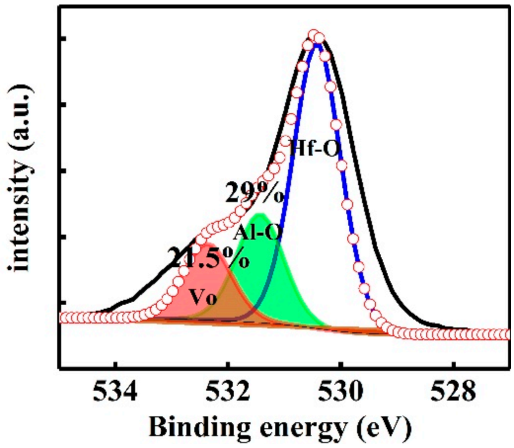

Figure S1. show the peal intensities of Al-O、Hf-O and oxygen vacancies in the O 1s spectrum with the Hf/Al cycle ratio of 34:1. We can see that the oxygen vacancy reach 21.5%.

Academic Editor(s): Name

Received: date
Revised: date
Accepted: date
Published: date

Copyright: © 2026 by the authors. Submitted for possible open access publication under the terms and conditions of the Creative Commons Attribution (CC BY) license.

S2 C-V curves of the devices at 4 V

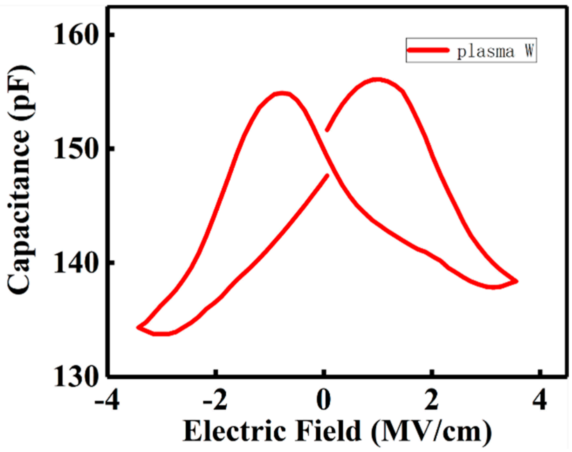

Figure S2. displays the C-V curves with bias voltages ranging from -4 to +4 V at 1KHZ.

S3 The P-E hysteresis curves of the hafnium-based thin films with 21.5% oxygen vacancies.

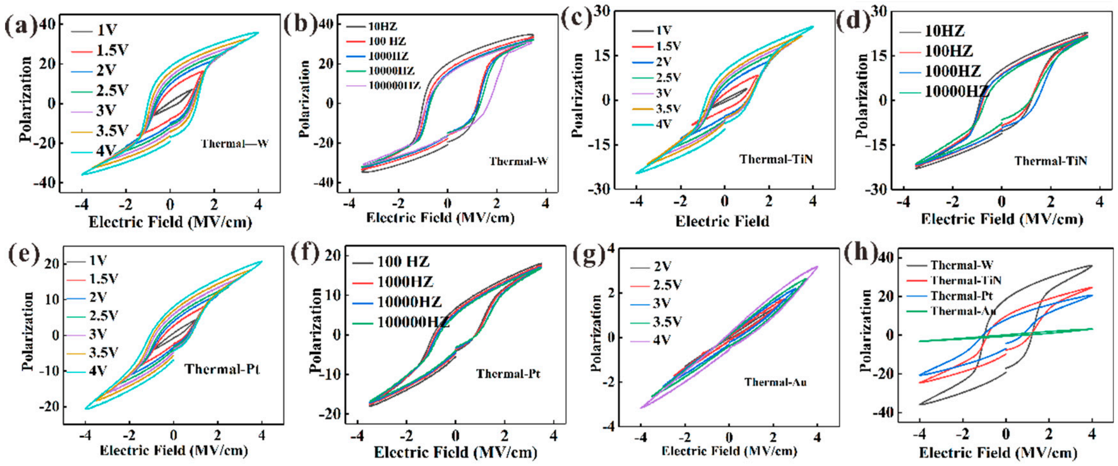

Figure S3. (a) shows the P-E hysteresis curves of the hafnium-based thin films of 21.5% oxygen vacancies with W top electrode under different voltage. FigS3 (b) shows the P-E hysteresis curves of the hafnium-based thin films of 21.5% oxygen vacancies with W top electrode under different frequency. FigS3 (c) shows the P-E hysteresis curves of the hafnium-based thin films of 21.5% oxygen vacancies with TiN top electrode under different voltage. FigS3 (d) shows the P-E hysteresis curves of the hafnium-based thin films of 21.5% oxygen vacancies with TiN top electrode under different frequency. FigS3 (e) shows the P-E hysteresis curves of the hafnium-based thin films of 21.5% oxygen vacancies with Pt top electrode under different voltage. FigS3 (f) shows the P-E hysteresis curves of the hafnium-based thin films of 21.5% oxygen vacancies with Pt top electrode under different frequency. FigS3 (g) shows the P-E hysteresis curves of the hafnium-based thin films of 21.5% oxygen vacancies with Au top electrode under different voltage. From the Figure S3(h), the devices with W top electrodes show the saturated P-E hysteresis curves, i.e., the remanent polarization of about 20 μC/cm^2.

S4 the remanent polarization and coercive field change with the top electrode changing

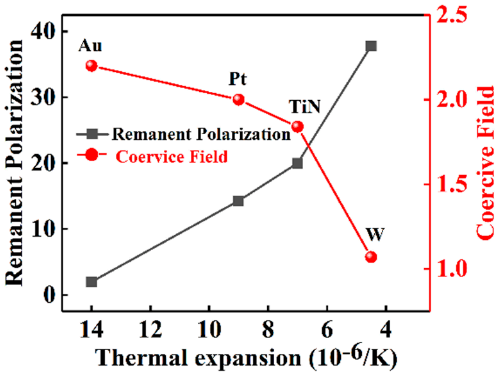

Figure S4. shows that the remanent polarization is increasing with the decreasing of thermal expansion. However, the coercive field is decreasing with the decreasing of thermal expansion.

S5The P-E hysteresis curves of the hafnium-based thin films with 14% oxygen vacancies.

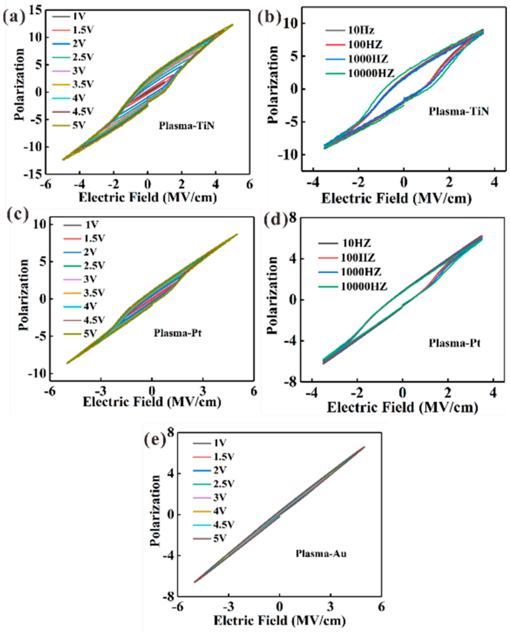

Figure S5. a) shows the P-E hysteresis curves of the hafnium-based thin films of 14% oxygen vacancies with TiN top electrode under different voltage. FigS5(b) shows the P-E hysteresis curves of the hafnium-based thin films of 14% oxygen vacancies with TiN top electrode under different frequency. FigS5 (c) shows the P-E hysteresis curves of the hafnium-based thin films of 14% oxygen vacancies with Pt top electrode under different voltage. FigS5 (d) shows the P-E hysteresis curves of the hafnium-based thin films of 14% oxygen vacancies with Pt top electrode under different frequency. FigS5 (e) shows the P-E hysteresis curves of the hafnium-based thin films of 14% oxygen vacancies with Au top electrode under different voltage.

S6 the retention characteristics test of hafnium-based thin films with 14% oxygen vacancies.

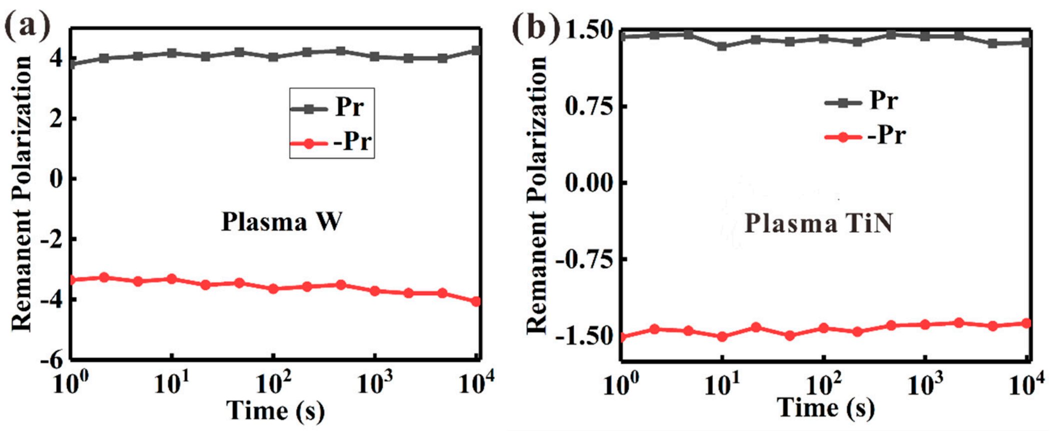

Figure S6. The retention time hold over 10^4 s for the ferroelectric devices with W top electrodes, as shown in Figure S6(a). Similarly, Figure S6(b) shows that the retention time is also over 10^4 s.
